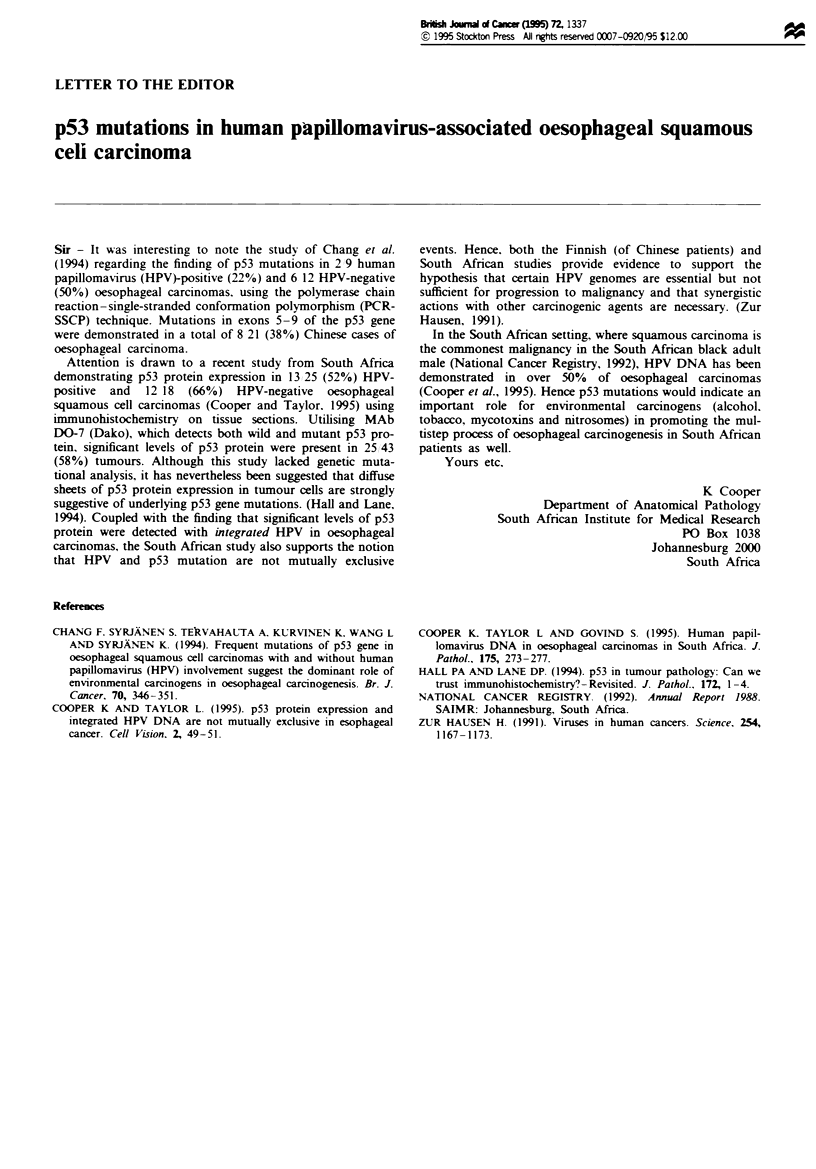# p53 mutations in human papillomavirus-associated oesophageal squamous cell carcinoma.

**DOI:** 10.1038/bjc.1995.511

**Published:** 1995-11

**Authors:** K. Cooper


© 1995 Stockton Press All rights reserved 0007-0920/95 $12.00

LETTER TO THE EDITOR

### p53 mutations in human papillomavirus-associated oesophageal squamous celi carcinoma

**Sir** - It was interesting to note the study of Chang *et al.* (1994) regarding the finding of p53 mutations in 2/9 human papillomavirus (HPV)-positive (22%) and 6/12 HPV-negative (50%) oesophageal carcinomas, using the polymerase chain reaction-single-stranded conformation polymorphism (PCR-SSCP) technique. Mutations in exons 5-9 of the p53 gene were demonstrated in a total of 8/21 (38%) Chinese cases of oesophageal carcinoma.

Attention is drawn to a recent study from South Africa demonstrating p53 protein expression in 13/25 (52%) HPV-positive and 12/18 (66%) HPV-negative oesophageal squamous cell carcinomas (Cooper and Taylor, 1995) using immunohistochemistry on tissue sections. Utilising MAb DO-7 (Dako), which detects both wild and mutant p53 protein, significant levels of p53 protein were present in 25/43 (58%) tumours. Although this study lacked genetic mutational analysis, it has nevertheless been suggested that diffuse sheets of p53 protein expression in tumour cells are strongly suggestive of underlying p53 gene mutations. (Hall and Lane, 1994). Coupled with the finding that significant levels of p53 protein were detected with *integrated* HPV in oesophageal carcinomas, the South African study also supports the notion that HPV and p53 mutation are not mutually exclusive events. Hence, both the Finnish (of Chinese patients) and South African studies provide evidence to support the hypothesis that certain HPV genomes are essential but not sufficient for progression to malignancy and that synergistic actions with other carcinogenic agents are necessary. (Zur Hausen, 1991).

In the South African setting, where squamous carcinoma is the commonest malignancy in the South African black adult male (National Cancer Registry, 1992), HPV DNA has been demonstrated in over 50% of oesophageal carcinomas (Cooper *et al.*, 1995). Hence p53 mutations would indicate an important role for environmental carcinogens (alcohol, tobacco, mycotoxins and nitrosomes) in promoting the multistep process of oesophageal carcinogenesis in South African patients as well.

Yours etc,

K Cooper
Department of Anatomical Pathology
South African Institute for Medical Research
PO Box 1038
Johannesburg 2000
South Africa

**References**

CHANG F, SYRJÄNEN S, TERVAHAUTA A, KURVINEN K, WANG L AND SYRJÄNEN K. (1994). Frequent mutations of p53 gene in oesophageal squamous cell carcinomas with and without human papillomavirus (HPV) involvement suggest the dominant role of environmental carcinogens in oesophageal carcinogenesis. *Br. J. Cancer*, **70**, 346-351.

COOPER K AND TAYLOR L. (1995). p53 protein expression and integrated HPV DNA are not mutually exclusive in esophageal cancer. *Cell Vision*, **2**, 49-51.

COOPER K, TAYLOR L AND GOVIND S. (1995). Human papillomavirus DNA in oesophageal carcinomas in South Africa. *J. Pathol.*, **175**, 273-277.

HALL PA AND LANE DP. (1994). p53 in tumour pathology: Can we trust immunohistochemistry?-Revisited. *J. Pathol.*, **172**, 1-4.

NATIONAL CANCER REGISTRY. (1992). *Annual Report 1988*. SAIMR: Johannesburg, South Africa.

ZUR HAUSEN H. (1991). Viruses in human cancers. *Science*, **254**, 1167-1173.